# Focal neurological deficits from multiple thromboembolic stroke complicating COVID-19 and limitations of stroke management during outbreak in Korea

**DOI:** 10.1007/s10072-021-05575-7

**Published:** 2021-08-25

**Authors:** Soo-Hyun Park, Woo Chang Chun

**Affiliations:** 1 Department of Neurology and Critical Care Medicine, Department of Hospital Medicine, Inha University Hospital, 27, Inhang-ro, Jung-gu, Incheon, Republic of Korea; 2 Department of Neurology, Inha University Hospital, Incheon, Republic of Korea

Dear Editor:

We are still fighting the new coronavirus disease 2019 (COVID-19) worldwide. Overall, 36% of patients with COVID-19 develop neurological symptoms [1]. Recent studies reported that the virus invades the central or peripheral nervous system via various mechanisms such as angiotensin-converting enzyme-2 receptors, blood–brain barrier injury, and immune injury [2]. Ischemic stroke can occur as a result of the virus penetrating the central nervous system. COVID-19 was associated with severe stroke symptoms due to large vessel strokes [3]. However, the etiology, mechanism, and treatment of stroke after the COVID-19 diagnosis have not been resolved. Our case experienced a strong association between multiple thromboembolic stroke and COVID-19, perhaps by triggering inflammation. We also consider the problems associated with acute ischemic stroke diagnosis and management during the COVID-19 pandemic.

An 82-year-old woman with known diabetes mellitus and hypertension was admitted to a tertiary hospital with fever (38.0 °C), cough, and diarrhea for 2 days. One week before hospitalization, her daughter was diagnosed with COVID-19. At admission, her chest X-ray showed multifocal infiltrates in both the lungs and chest computed tomography (CT) showed peripherally distributed patchy ground-glass opacification. Nasopharyngeal swab tested positive for severe acute respiratory syndrome coronavirus 2 (SARS-CoV-2) on real-time reverse transcription-polymerase chain reaction assay. Laboratory findings revealed elevated C-reactive protein level (3.76 mg/dL; normal 0.0–0.5 mg/dL), elevated D-dimer level (16.67 mg/mL; normal 0.0–0.5 μg/mL), normal leukocyte count (7420/mm^3^; normal 4000–10,000/mm^3^), and normal prothrombin time (12.7 s; normal 11.0–15.0 s). Initial treatment involved oral lopinavir/ritonavir (400 mg/1000 mg) and hydroxychloroquinine (400 mg), and subcutaneous low molecular weight heparin (40 mg). However, the fever persisted, and her chest X-ray findings worsened. Intravenous immunoglobulin (0.3 g/kg) was added, including oxygen (2 L/min) via nasal prongs.

On the 8th day of hospitalization (11 days after COVID-19), the patient abruptly complained of weakness and numbness in the right arm. The neuro-intensivist was mobilized to the COVID-19 isolation unit. The initial National Institutes of Health Stroke Scale score was 2, with a power grade of 4 + and decreased sensation in the right arm. The other neurological examinations were normal. Brain CT showed a focal low-density lesion in the right cerebellum (Fig. 1a). The patient’s symptoms were mild, and no intravenous alteplase was administered. Brain magnetic resonance image showed multiple high signal intensity lesions with low apparent diffusion coefficient value in the right cerebellum (Fig. 1b), left precentral gyrus (Fig. 1c), and left frontoparietal cortex (Fig. 1d). Brain magnetic resonance angiography showed no steno-occlusive lesions (Fig. 1e). The patient was transferred to an isolation unit for neurological monitoring. The main lesion of the left precentral gyrus caused weakness and numbness of the right arm. Aspirin, clopidogrel, and statin were added to treatment for ischemic stroke. However, the acute diagnosis and treatment of ischemic stroke were delayed by about 23 h from symptom onset to when the first brain imaging was performed. Trans-thoracic echocardiography (TTE) and 24-h Holter monitoring were also performed separately later, but the results were normal. Eventually, the patient’s neurological symptoms and COVID-19 symptoms improved, and she was discharged.

**Fig. 1:**
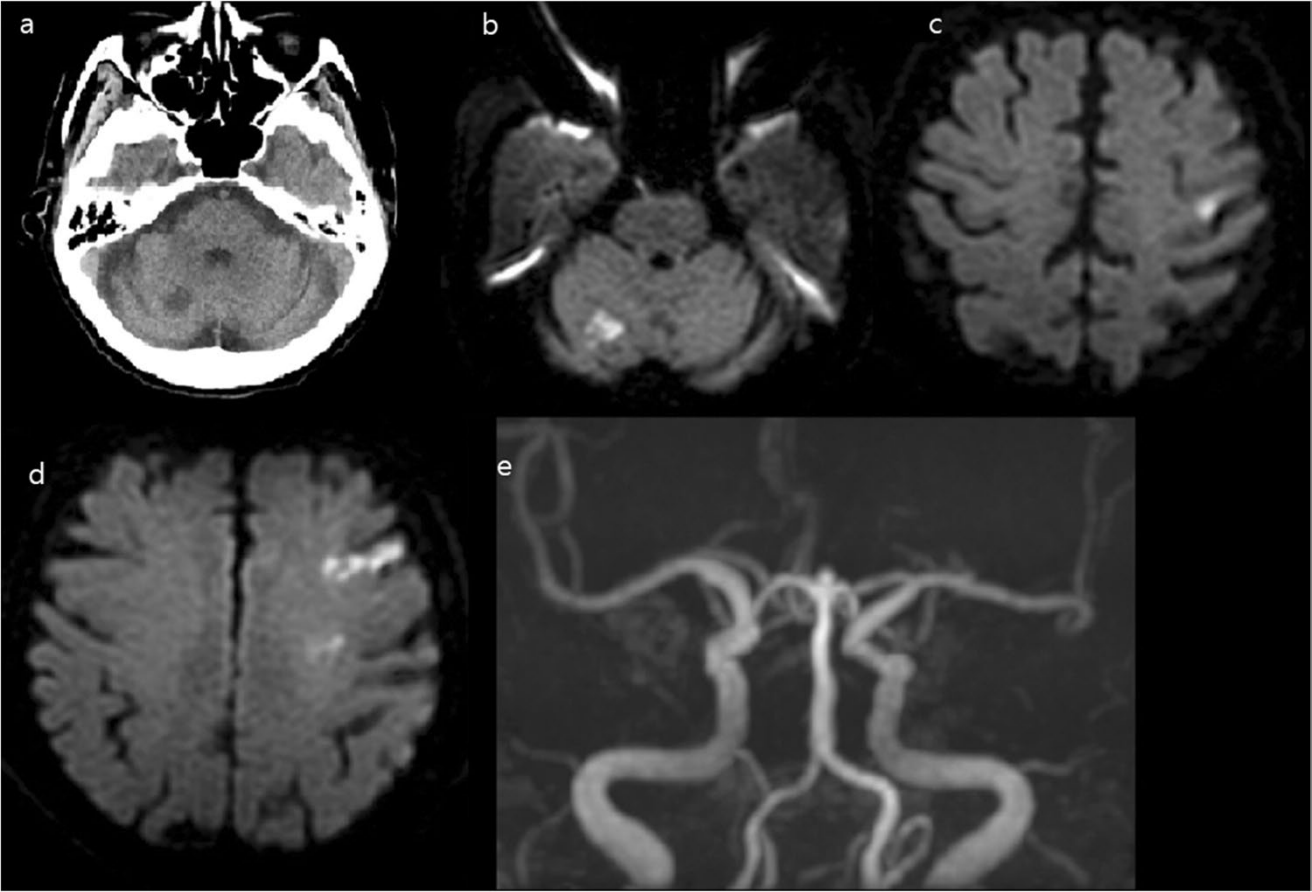
Radiologic findings of the patient. **A** Non-contrast-enhanced brain CT showing a focal low-density lesion in the right cerebellum. **B** Diffuse-weighted brain MRI showing diffuse high signal intensity in the right cerebellum. **C** Diffuse-weighted brain MRI showing diffuse high signal intensity in the left precentral gyrus. **D** Diffuse-weighted brain MRI showing diffuse high signal intensity in the left frontoparietal cortex. **E** Brain magnetic resonance angiography showing luminal irregularity in the basilar artery and mild segmental stenosis in the distal segment

Our case shows that COVID-19 can develop focal neurologic deficits. In addition, as a result of the invasion of the central nervous system, thromboembolic stroke can occur due to COVID-19, as confirmed on brain images and laboratory tests.

Recent studies report how SARS-CoV-2 and cerebrovascular disease might be related [4]. Elevated CRP and D-dimer levels may be the main cause of stroke in COVID-19 patients as they represent an active inflammatory state and abnormalities of the coagulation pathway [5]. These mechanisms activate cytokines to produce thrombin, resulting in thromboembolism and elevated D-dimer levels. Our patient also had elevated CRP and D-dimer levels and had no apparent abnormalities in other tests for stroke mechanism. Although the occurrence of stroke due to microvascular thrombosis is becoming more evident, recent studies have shown that a significant number of COVID-19 patients develop large vascular occlusions with severe stroke symptoms [3, 4]. However, according to our case, COVID-19 can also represent focal neurologic deficits. Our data alone cannot determine the causal relationship between COVID-19 and stroke and the severity of stroke symptoms; thus, a large number of data are needed. Nevertheless, our case provides early insights into stroke care in COVID-19 patients.

Stroke has been reported in about 6% of COVID-19 patients [1]. Similar to what is known in other studies, focal neurological symptoms occurred after our patient had been managed for COVID-19 for 11 days [3–5]. However, the patient’s neurological examination proceeded relatively quickly, but the diagnosis and treatment of ischemic stroke was delayed as a long time was taken to perform the first brain imaging and cardiac function evaluation. Because of the focus on the treatment of COVID-19, the stroke evaluation in our case was performed slowly and inappropriately. In fact, our patient did not undergo brain imaging and TTE immediately after exhibiting stroke symptoms. This is longer than reported by the Clinical Research Collaboration for Stroke in Korea statistics in 2018. Furthermore, there were restrictions on rehabilitation treatment.

Attending physicians and patients wear protective equipment such as eye protection, gloves, and gowns to prevent the spread of COVID-19. The medical space for COVID-19 patients is highly compartmentalized. This makes it difficult for stroke patients having COVID-19 to immediately recognize stroke symptoms due to fear of spreading the virus. Treatment for COVID-19 is very important, but stroke evaluation and management should not be ignored.

Recently, recommendations for the diagnosis and treatments of stroke patients during the COVID-19 pandemic have been offered. However, with the small number of centers treating COVID-19 and patients actually receiving COVID-19 treatment, it is challenging to implement the recommendations. Therefore, we need a large sample size to confirm the recommendations. The pre-hospital process to hospital delivery may require several steps and can be complex during the COVID-19 outbreak. In addition, it is difficult to manage the behavior of acute stroke patients with COVID-19 after stroke symptoms occur. Therefore, hospitals and governments should consider the recommendations for the pre-hospital delivery process, in-hospital diagnosis, and treatments to care for stroke patients with confirmed COVID-19, including general patients with acute stroke. In addition, urgent support networks may be required to provide education on stroke for COVID-19 patients.

This study had some limitations. It was based on data from only one tertiary hospital in South Korea. Stroke care systems may differ among hospitals. The prevalence and treatment network for COVID-19 varies among countries. Therefore, the behavior and awareness of stroke patients during the COVID-19 outbreak may also differ. However, the international community agrees on the proper response to COVID-19. According to our study, there are common issues that need to be addressed in patients diagnosed with COVID-19 and stroke.

In conclusion, if COVID-19 patients have neurological symptoms and elevated D-dimer levels, as it is the marker of inflammation, clinicians should be aware of the possibility of stroke occurrence. We need to note that COVID-19 can appear as a local neurological defect, and the diagnosis and treatment methods for stroke patients caused by COVID-19 need to be re-evaluated.

## Data Availability

All data generated or analyzed during this study are included in this published article.
